# PKC Activation Induces Inflammatory Response and Cell Death in Human Bronchial Epithelial Cells

**DOI:** 10.1371/journal.pone.0064182

**Published:** 2013-05-17

**Authors:** Hyunhee Kim, Ricardo Zamel, Xiao-Hui Bai, Mingyao Liu

**Affiliations:** 1 Latner Thoracic Surgery Research Laboratories, Toronto General Research Institute University Health Network, Toronto, Ontario, Canada; 2 Department of Physiology, Faculty of Medicine, University of Toronto, Toronto, Ontario, Canada; 3 Departments of Surgery and Medicine, Institute of Medical Science, Faculty of Medicine, University of Toronto, Toronto, Ontario, Canada; National Jewish Health, United States of America

## Abstract

A variety of airborne pathogens can induce inflammatory responses in airway epithelial cells, which is a crucial component of host defence. However, excessive inflammatory responses and chronic inflammation also contribute to different diseases of the respiratory system. We hypothesized that the activation of protein kinase C (PKC) is one of the essential mechanisms of inflammatory response in airway epithelial cells. In the present study, we stimulated human bronchial lung epithelial (BEAS-2B) cells with the phorbol ester Phorbol 12, 13-dibutyrate (PDBu), and examined gene expression profile using microarrays. Microarray analysis suggests that PKC activation induced dramatic changes in gene expression related to multiple cellular functions. The top two interaction networks generated from these changes were centered on NFκB and TNF-α, which are two commonly known pathways for cell death and inflammation. Subsequent tests confirmed the decrease in cell viability and an increase in the production of various cytokines. Interestingly, each of the increased cytokines was differentially regulated at mRNA and/or protein levels by different sub-classes of PKC isozymes. We conclude that pathological cell death and cytokine production in airway epithelial cells in various situations may be mediated through PKC related signaling pathways. These findings suggest that PKCs can be new targets for treatment of lung diseases.

## Introduction

Airway epithelial cells are in the front line of host defence against various airborne pathogens, allergens, and irritants [1]. Airway epithelium functions as a physical barrier with secretion and clearance functions. In addition, airway epithelial cells can produce cytokines and chemokines to initiate local inflammatory and immune response. After airway injury, epithelial cell migration occurs as an early mechanism of repair, and this is mediated by cytokines and growth factors. Subsequent cell proliferation and differentiation restore the damaged epithelium [2]. Malfunction of cellular responses may lead to various chronic inflammatory diseases, such as asthma and chronic obstructive pulmonary disease [1].

The inflammatory responses of airway epithelial cells are an important component in innate immunity. However, excessive inflammatory responses can lead to cell death and tissue damage, and ultimately, chronic inflammation may contribute to the pathogenesis of airway diseases. Thus, inflammatory responses require precise regulation mediated by multiple intracellular signal transduction pathways [3].

Protein Kinase C (PKC) is a well-known family of homologous serine threonine kinases with a prominent role in many cellular functions. A total of 15 isozymes of PKC have been identified, and they are subsequently classified into 3 general subfamilies depending on their specific mode of activation: classical, novel, and atypical [4]. Pulmonary insult by various harmful substances can lead to activation of multiple PKC subtypes in airway epithelial cells. For example, in pulmonary epithelial cells exposed to asbestos, a carcinogen, PKCδ is activated and translocated to the nucleus [5]. One study suggests that cell death induced by asbestos is PKCδ-dependent [6]. PKC activation can induce dramatic morphological changes of human bronchial epithelial cells that lead to podosome formation, which is followed by secretion of matrix metalloproteases and alteration in cell motility [7], [8]. Cigarette smoking induces IL-8 production and inhibition of ciliary motility in airway epithelial cells via PKC activation [9], [10]. PKCα expression is noticeably high in the airway of COPD patients [11]. In the case of asthma, β-adrenergic receptor expression is increased by IL-1β stimulation and this is mediated by PKC [12].

These experimental and clinical observations suggest that PKC activation could be one of the essential mechanisms regulating the response of airway epithelial cells when they are stimulated by a variety of injurious factors. In this study, we hypothesized that PKC activation has profound effects on human airway epithelial cells through gene expression, cell proliferation, cell survival, motility, and especially the activation of inflammatory responses. We examined the effect of direct PKC activation in airway epithelial cells on gene expression using microarray, and further studied its impacts on the cell death and production of inflammatory mediators at both gene and protein levels.

## Materials and Methods

### Cell line and reagents

Human bronchial epithelial cell line (BEAS-2B) was obtained from ATCC (Manassas, VA) [7], [8], [13]. Cells are cultured in low-glucose Dulbecco's modified Eagle's medium (DMEM) with 10% fetal bovine serum (FBS; GIBCO, Carlsbad, CA) [14]. Cells were grown at 37°C with 5% CO_2_. Phorbol 12,13-dibutyrate (PDBu), a PKC activator, was purchased from Sigma Aldrich (St. Louis, MO). Bisindolylmaleimide I (BIM-1), Ro-31-8220, GÖ6976, and rottlerin were purchased from EMD Bioscience (Darmstadt, Germany) [7].

### SiRNA

PKCα, PKCδ, and scramble siRNA (Santa Cruz Biotechnology, Santa Cruz, CA) were transfected into BEAS-2B cells using Oligofectamine (Invitrogen, Carlsbad, CA), as we previously described [7], [13]. Cells were plated in 6 well plates at concentration of 150,000 cells per well. Each of the wells had 50 nM of siRNA and 10 µM of Oligofectamine, diluted in optiMEM. After the transfection, cells were incubated for 24 hours, washed and incubated again in DMEM with 10% fetal bovine serum for another 48 hours before PDBu stimulation.

### Microarray

The mRNA expression profile in BEAS-2B cells was investigated with microarray, as we previously described [15]. Three groups were prepared: control, 0.5 hour of PDBu stimulation, and 4 hours of PDBu stimulation. Each group consisted of three biological replicates. RNeasy kit (Qiagen, Valencia, CA) was used to extract total RNA. High-Capacity cDNA Reverse Transcription kits (Applied Biosystems, Foster City, CA) and PTC-100™ Programmable Thermal controller (MJ Research Inc., Watertown, MA) were used to synthesize cDNA. The RNA Integrity Number (RIN) was determined by Agilent Bioanalyzer 2100 (Agilent Technologies, Inc., Santa Clara, CA). The microarray used was Human Gene ST 1.0, containing 28,132 probe sets from Affymetrix (Santa Clara, CA). Affymetrix CEL files were imported and analyzed using Partek software (Partek Inc., St. Louis, MO). Microarray data were pre-processed using the robust multi-array average (RMA) technique. Principle Component Analysis (PCA), Hierarchical cluster analysis, and differential expression analysis (ANOVA) were performed in Partek. Benjamini-Hochberg false discovery rate adjustment was used to correct for multiple testing. Interaction networks were generated using Ingenuity Pathway Analysis (IPA; Ingenuity Systems, Inc., Redwood City, CA). Gene-annotation enrichment analysis was performed using DAVID Functional Annotation bioinformatics analysis (National Institute of Allergy and Infectious Diseases, NIH). The microarray data has been deposited in the Gene Expression Omnibus (GEO) repository (accession number GSE44747).

### Cell viability assay

BEAS-2B cells were cultured in a 6-well plate at 300,000 cells per well for 24 hours. The cells were then treated with or without PDBu, trypsinized and resuspended in the culture medium. Viable cells were counted with a Sceptor 2.0 Handheld Automated Cell Counter (EMD Millipore, Billerica, MA).

### Quantitative RT-PCR

The primers used for quantitative reverse transcription were as shown in Table S1. The total RNA was extracted using TRIzol Reagent (Invitrogen, Carlsbad, CA). qRT-PCR was performed with 2×QuantiTect SYBR Green PCR kit (Qiagen, Mississauga, Canada) on LightCycler480 (Roche, Mannheim, Germany) as previously described [15]–[17]. Each assay had a standard curve of five serial dilutions and a no-template negative control.

### Measurement of soluble cytokines and chemokines in culture medium

After cells were stimulated with PDBu for 0.5 or 4 hours, culture medium was collected, centrifuged at 10,000 rpm, and the supernatant was stored at −80°C. Cytokines, chemokines and growth factors in the culture medium were quantified using MILLIPLEX MAP Human Cytokine/Chemokine - Premixed 42 Plex (Category number: MPXHCYTO60KPMX42). Four replicates were tested in each of the groups. Concentrations of IL-6, IL-8, G-CSF, and GRO-1α in culture medium were also quantified with DuoSet®ELISA systems (R&D Systems, Minneapolis, MN). The medium was diluted either by 1∶5 or by 1∶10 to fit the standard curve of the kit. The assay was performed following the manufacturer's instructions.

### Western Blots and PKC Translocation assays

These experiments were performed according to the standard protocols as we previously described [7], [14], [18].

### Statistical tests

Student-t test and analysis of variance (ANOVA) with linear contrasts, followed by post hoc analysis (Tukey's range test), were used to compare the means of two or more groups. The significance cutoff was set to p≤0.05. The values are denoted as mean ± S.D. GraphPad Prism 5 software was used to calculate statistics and produce graphs.

## Results

### PKC activation results in significant changes in gene expression profile

**Figure 1:**
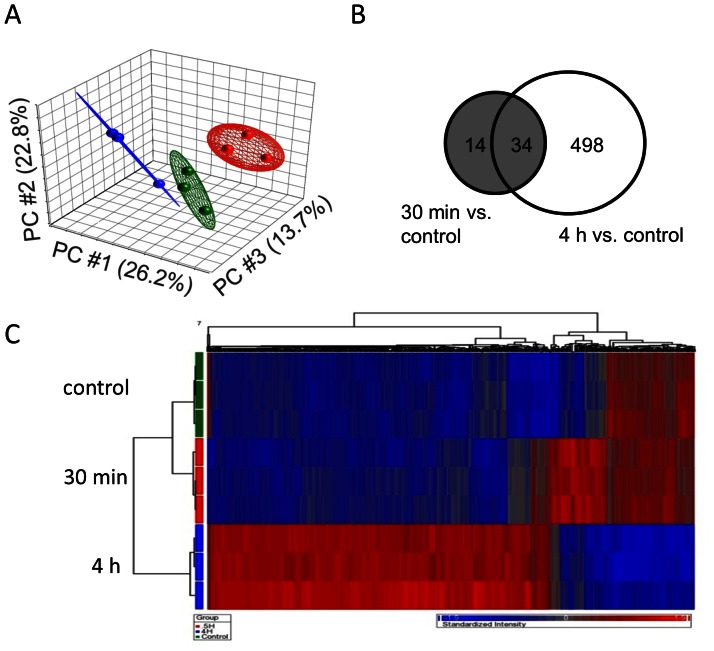
PKC activation-induced gene expression profiles. (A) PCA analysis clearly separates the three groups: control (red), 0.5 hours of PDBu stimulation (blue), and 4 hours of PDBu stimulation (green). (B) Venn diagram shows the differentially regulated genes between the two time points. (C) Hierarchical clustering analysis clustering analysis of differentially expressed genes automatically segregated the three groups, and most of the genes were up-regulated by PKC activation.

**Figure 2:**
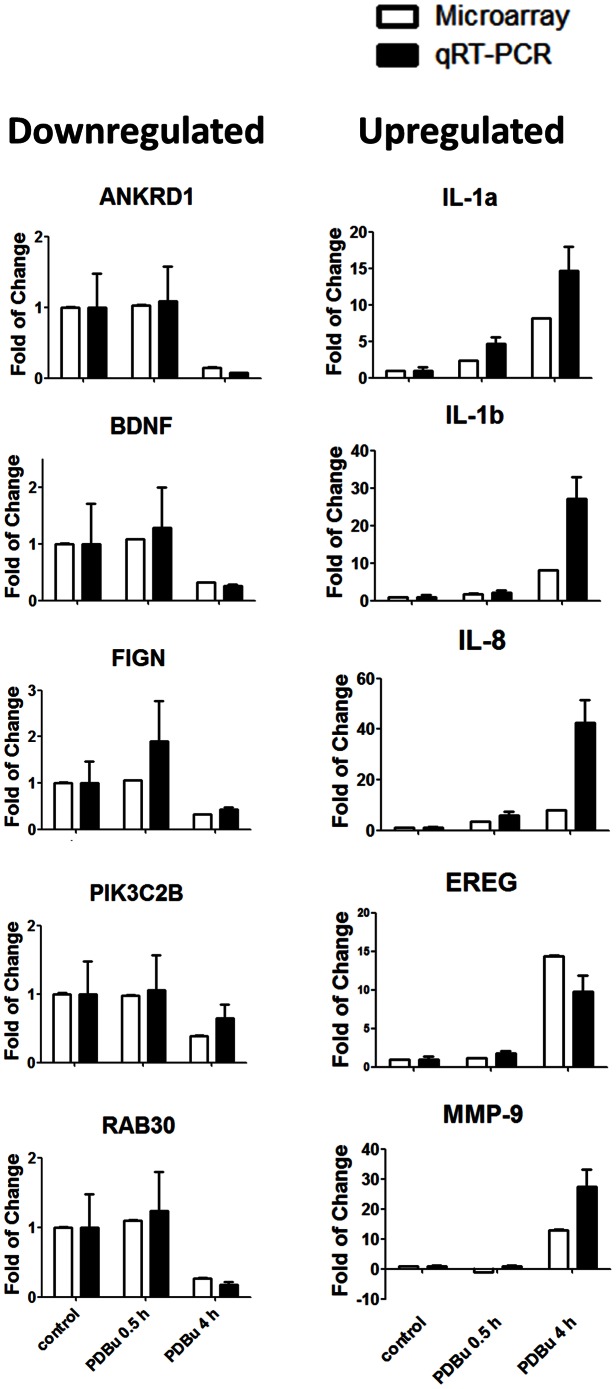
Validating microarray results with quantitative RT-PCR analysis. Five down-regulated (ANKRD1, BDNF, FIGN, PIK3C2B, and RAB30) and five up-regulated genes (EREG, IL-1a, IL-1b, IL-8, MMP-8) were selected from the microarray data and their expression was examined with qRT-PCR. The trends of which the genes are up and down-regulated are similar between microarray and qRT-PCR results. The PCR results were normalized to a housekeeping gene, SDHA.

To determine the overall effects of PKC activation on human bronchial epithelial cells, we first stimulated BEAS-2B cells with PDBu (500 nM, a dose selected from our previous studies) [7], [8]. PDBu treatment induced translocation of PKCα from cytosol to membrane fractions, and increased phosphorylation of other PKC isoforms in a time-dependent manner (Figure S1). Overall gene expression changes by PKC activation were examined using microarray. We analyzed differences between three groups: control (no PDBu stimulation), 0.5 hours and 4 hours of PDBu stimulation. Principle Component Analysis (PCA) demonstrated that each of the groups is clearly distinct from each other (Figure 1A). We used ANOVA with linear contrasts for analysis of differential gene expression. We defined genes as significantly changed by false discovery rate (FDR) less than or equal to 5% and fold-change greater than 1.3. Upon 0.5 hours of PKC activation, 48 genes were significantly up-regulated, and they are mainly early response genes (e.g. Early growth response 1, 2, and 3, IER2, Fos, JunB, FosB), and cytokines (e.g. CXCL2, IL-8, IL-6, PTX3, CXCL1) (Table 1). Furthermore, 532 genes were significantly changed after 4 hours of the PKC activation. The Venn-diagram shown in Figure 1B indicates that 34 of the 48 changed genes after 0.5 hours remained significantly changed after 4 hours. Hierarchical cluster analysis further demonstrates that PKC activation has a significant impact on the gene expression profile in a time-dependent manner (Figure 1C). Most of the genes modulated after 4 hours of PDBu stimulation were also up-regulated, as shown in the heat map (Figure 1C). Five significantly up-regulated and down-regulated genes indicated by microarray were validated with RT-PCR. All of them showed similar changes between RT-PCR and microarray data (Figure 2). The top 20 most significantly changed genes after 4 hours of PDBu stimulation are listed in Table 2. Overall, PKC activation induced significant changes in the gene expression profile.

**Table 1:** The top 20 genes that are significantly changed by 0.5 h PDBu treatment.

gene name	Gene Symbol	RefSeq	p-value	Fold-Change
early growth response 1	EGR1	NM_001964	0.0003	12.768
early growth response 3	EGR3	NM_004430	0.0065	8.316
early growth response 2	EGR2	NM_000399	0.0006	8.212
murine osteosarcoma viral oncogene homolog	FOS	NM_005252	0.0020	6.765
prostaglandin-endoperoxide synthase 2	PTGS2	NM_000963	0.0006	5.153
zinc finger protein 36, C3H type, homolog	ZFP36	NM_003407	0.0003	4.031
chemokine (C-X-C motif) ligand 2	CXCL2	NM_002089	0.0056	3.461
interleukin 8	IL8	NM_000584	0.0044	3.352
dual specificity phosphatase 1	DUSP1	NM_004417	0.0044	3.193
interleukin 6 (interferon, beta 2)	IL6	NM_000600	0.0069	3.151
hairy and enhancer of split 1	HES1	NM_005524	0.0011	3.143
immediate early response 2	IER2	NM_004907	0.0069	3.130
transmembrane protein 88	TMEM88	NM_203411	0.0034	3.104
jun B proto-oncogene	JUNB	NM_002229	0.0003	3.099
dual specificity phosphatase 5	DUSP5	NM_004419	0.0001	2.967
pentraxin-related gene	PTX3	NM_002852	0.0011	2.960
nuclear factor of kappa light polypeptide gene enhancer	NFKBIZ	NM_031419	0.0020	2.906
chemokine (C-X-C motif) ligand 1	CXCL1	NM_001511	0.0069	2.860
FBJ murine osteosarcoma viral oncogene homolog B	FOSB	NM_006732	0.0087	2.821
dual specificity phosphatase 6	DUSP6	NM_001946	0.0072	2.593

**Table 2:** The top 20 genes that are significantly changed by 4 h PDBu treatment.

Gene name	Gene Symbol	RefSeq	p-value	Fold-Change
Epiregulin	EREG	NM_001432	3.96E-06	14.33
matrix metallopeptidase 9	MMP9	NM_004994	6.72E-06	12.97
solute carrier family 16, member 6	SLC16A6	NM_004694	2.24E-05	10.47
prostaglandin-endoperoxide synthase 2	PTGS2	NM_000963	2.28E-05	10.18
leukemia inhibitory factor	LIF	NM_002309	3.31E-06	10.00
dual specificity phosphatase 6	DUSP6	NM_001946	4.24E-05	9.36
glutamine-fructose-6-phosphate transaminase 2	GFPT2	NM_005110	1.67E-05	8.76
MST131	MST131	ENST00000423322	4.74E-05	8.18
interleukin 1, alpha	IL1A	NM_000575	6.95E-06	8.14
interleukin 1, beta	IL1B	NM_000576	1.27E-05	8.07
interleukin 8	IL8	NM_000584	7.06E-05	8.00
chemokine (C-C motif) ligand 2	CCL2	NM_002982	3.58E-04	7.68
serpin peptidase inhibitor, clade B	SERPINB2	NM_001143818	2.97E-06	7.61
amphiregulin	AREG	NM_001657	9.60E-05	7.38
inhibin, beta A	INHBA	NM_002192	1.25E-05	6.88
metastasis suppressor 1	MTSS1	NM_014751	3.20E-05	6.86
ankyrin repeat domain 1	ANKRD1	NM_014391	1.59E-04	-6.65
endothelial cell-specific molecule 1	ESM1	NM_007036	3.20E-05	6.51
colony stimulating factor 2 (granulocyte-macrophage)	CSF2	NM_000758	1.02E-03	6.48
early growth response 1	EGR1	NM_001964	5.31E-05	6.45

### PKC activation modulates genes involved in pathways related to multiple functions, especially inflammatory response and cell death

We then performed DAVID Functional Annotation bioinformatics analysis on genes regulated by 4 hours of PDBu stimulation. The top ten functional terms are shown in Table 3. The data was further investigated via Ingenuity Pathway Analysis (IPA). The top five biologically functional networks included (1) cell death, cellular growth, and proliferation, (2) gene expression, (3) cell cycle and cell to cell signaling, (4) cellular movements and (5) immune cell trafficking.

**Table 3:** DAVID Annotation showing top 10 enriched functional terms associated with PKC activation.

Term	Count	p value (Benjamini)
regulation of apoptosis	62	3.69E-12
response to wounding	49	1.99E-12
regulation of cell proliferation	58	5.53E-11
regulation of phosphate metabolic process	43	3.79E-10
regulation of cytokine production	26	8.18E-10
positive regulation of cellular biosynthetic process	51	1.06E-09
regulation of phosphorylation	41	1.23E-09
positive regulation of biosynthetic process	51	1.44E-09
anti-apoptosis	27	1.54E-09
positive regulation of developmental process	31	1.89E-09

**Figure 3:**
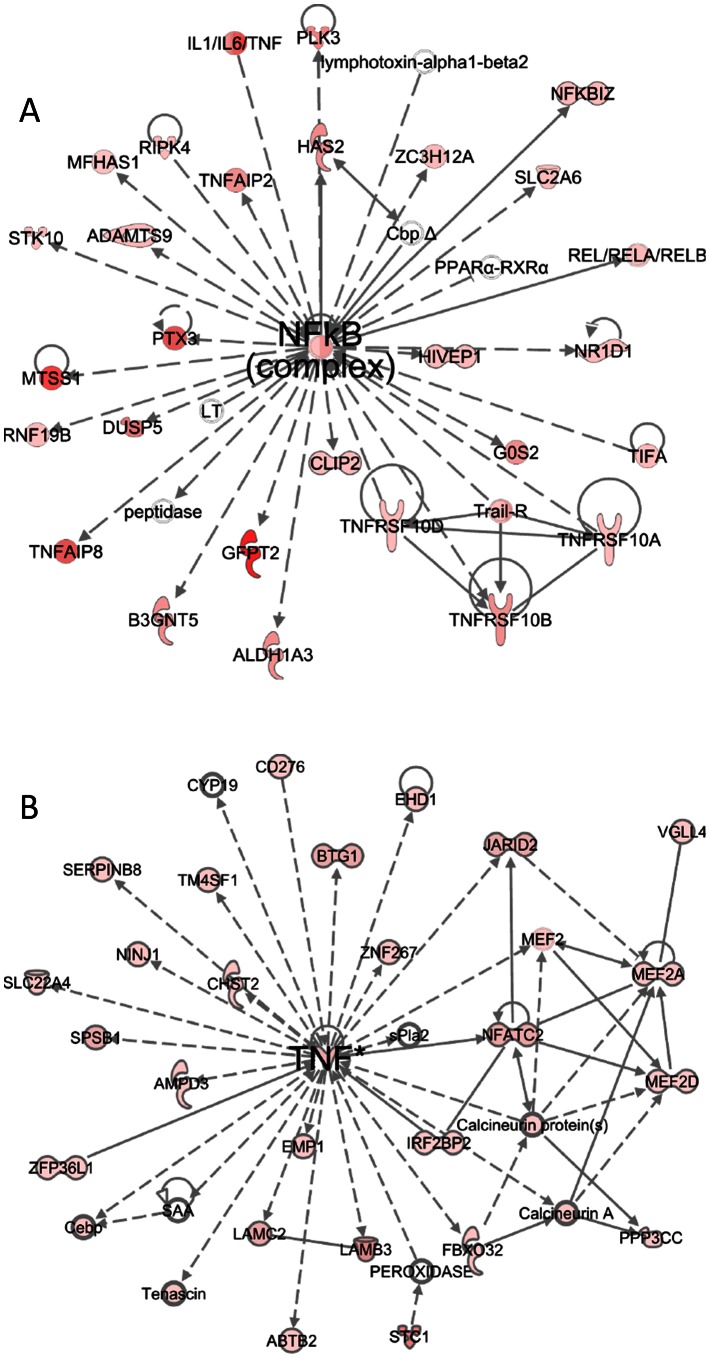
Top two IPA interaction networks suggested by microarray are NFκB and TNF-α. Top two interaction networks generated by Ingenuity Pathway Analysis from significantly regulated genes after 4 hours of PDBu stimulation. The networks were centered on NFκB (A) and TNF-α (B). Both of these networks are closely related to inflammation and cell death.

The top interaction network generated by IPA was centered on NFκB, which is shown to interact with multiple cytokines (e.g., IL-1, IL-6, TNF), TNFα induced proteins (e.g., PTX3), TNF receptor superfamily members, components of NFκB pathway, and receptors of inflammatory mediators (Figure 3A), suggesting that PKC activation may mediate inflammatory response signals through NFκB pathway. Indeed, increased IκBα degradation was noticed through western blotting after PDBu stimulation (data not shown). The second network that activation of PKC induced was centered on TNF-α (Figure 3B), implicating that TNFα induced biological signals may be mediated via PKC activation.

### PKC activation induces cell death in human lung epithelial cells

**Figure 4:**
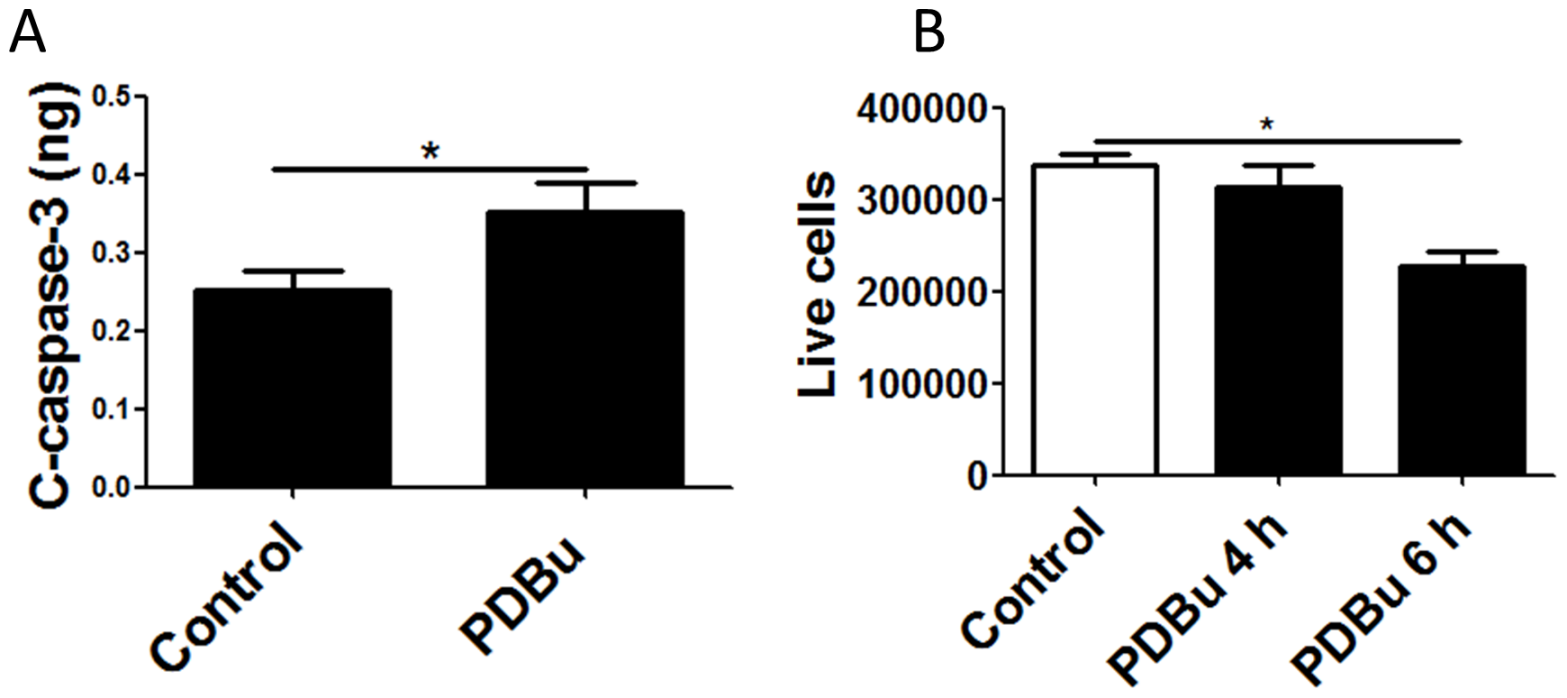
PKC activation induces cell death in lung epithelial cells. (A) The cleaved form of Caspase-3 is significantly increased after 4 hours of PDBu stimulation. (B) The number of live cells was decreased significantly after 6 hours.

Both functional annotation enrichment and interaction networks suggest that activation of PKC leads to the up-regulation of pathways related to inflammatory response and cell death. We further analyzed whether PKC activation can induce cell death in BEAS-2B cells, as inferred by the microarray data analysis. When the cells were treated with PDBu for 4 hours, the level of cleaved caspase-3, activated effector caspase, was significantly increased (Figure 4A). We further treated cells with PDBu and counted viable cells using an automatic cell counter. While PDBu treatment did not affect cell viability within 4 hours, it induced a significant reduction in cell viability after 6 hours (Figure 4B).

### PKC activation increases the gene expression and secretion of multiple cytokines in human lung epithelial cells

**Figure 5:**
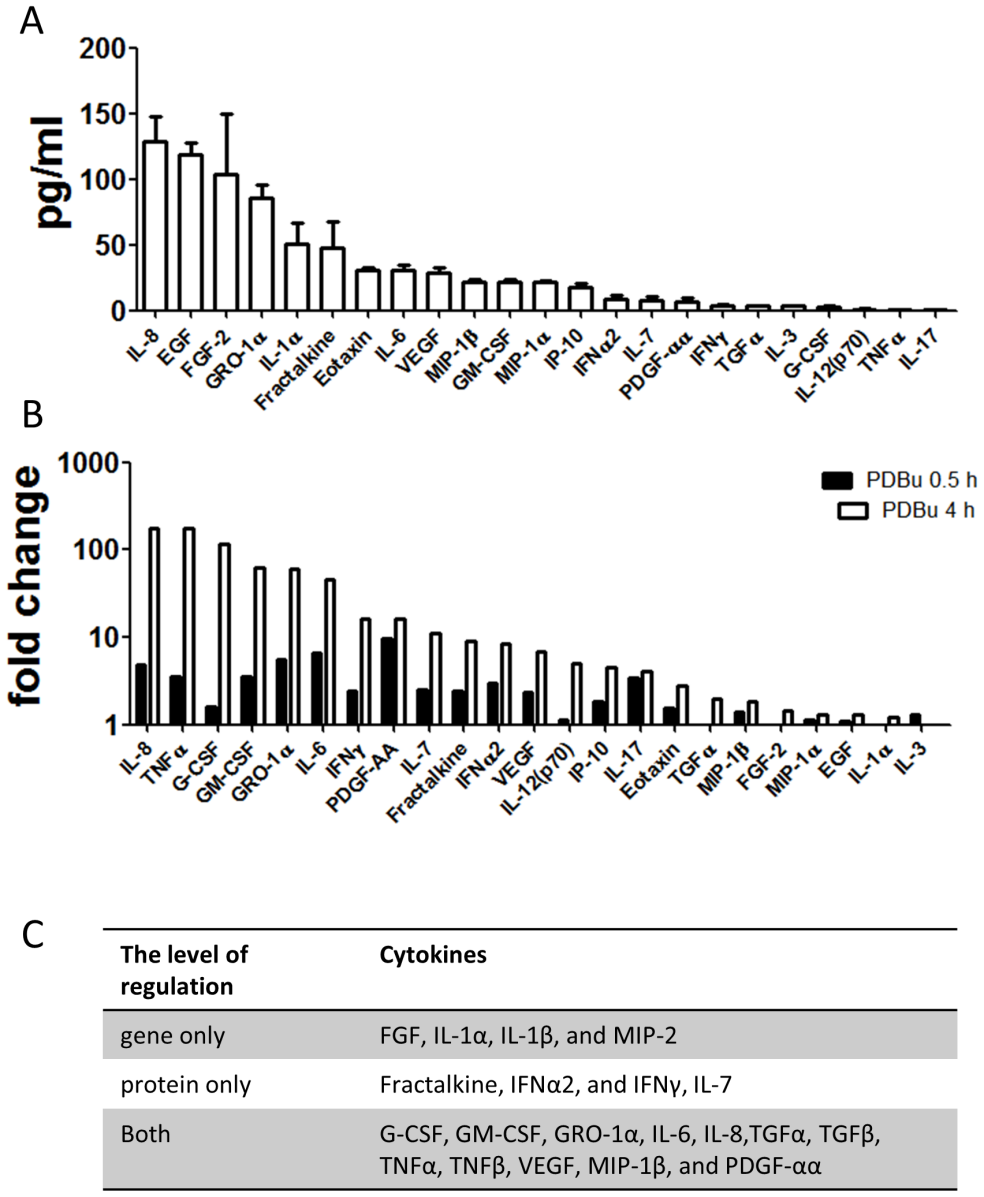
PKC activation increases the secretion of various cytokines. (A) The basal level of cytokines produced by BEAS-2B cells. Twenty-three cytokines were detected by Cytometric Beads Assay. (B) The fold-change of cytokines after PDBu stimulation at two different time points: 0.5 h and 4 h h. (C) These cytokines are differentially regulated at gene and/or protein levels, as determined by comparing the microarray and the cytometric beads data.

Microarray analysis suggested that the activation of inflammatory response and cytokine production occurs by PKC activation. Thus, we investigated the changes in production of chemokines and cytokines in BEAS-2B cells after PDBu treatment. To determine the overall cytokine profile, the cell-culture medium was screened with cytometric beads array (CBA) that simultaneously detects 42 human cytokines. Among them, 23 cytokines were detected in the culture medium of BEAS-2B cells under control condition (Figure 5A). PDBu stimulation increased production of cytokines at 0.5 hours, and many of them further increased after 4 hours. Fifteen of the 23 cytokines were significantly increased after 4 hours (Figure 5B).

We then compared the microarray analysis with the cytokine profile data from CBA, to determine whether the cytokines and chemokine were modulated at the mRNA and/or protein levels. We found that G-CSF, GM-CSF, GRO-1α, IL-6, IL-8, TGFα, TGFβ, TNFα, TNFβ, VEGF, MIP-1β, and PDGF-αα had increased both in mRNA and in protein levels in the culture medium after 4 hours of PDBu stimulation. This suggests that the cytokines were both synthesized and released following PKC activation. In contrast, Fractalkine, IFNα2, IFNγ, and IL-7 were increased in protein level, but not in mRNA level, suggesting that these cytokines were released in response to the PKC activation, but their gene expression was not increased upon PKC activation. Finally, FGF, IL-1α, IL-1β, and MIP-2 were significantly up-regulated in mRNA level, but were not elevated at protein levels. This suggests that the gene expression of these cytokines is up-regulated by PKC activation, but the protein synthesis and/or release may be PKC-independent. These finding are summarized in Figure 5C.

### PKC activation induced production of cytokines may be mediated by different sub-class of PKC isozymes

Both classical and novel PKC isozymes can be activated upon PDBu stimulation. To further dissect which subgroups of the isozymes are responsible for cytokine release, we incubated cells with PKC inhibitors 1 hour prior to PDBu stimulation. Bisindoylmaleimide-1 (Bim-1, 1 µM) and Ro31-8220 (1 µM) are pan PKC inhibitors, while GȌ6796 (1 µM) and rottlerin (1 µM) are specific classical and novel PKC inhibitors, respectively.

**Figure 6:**
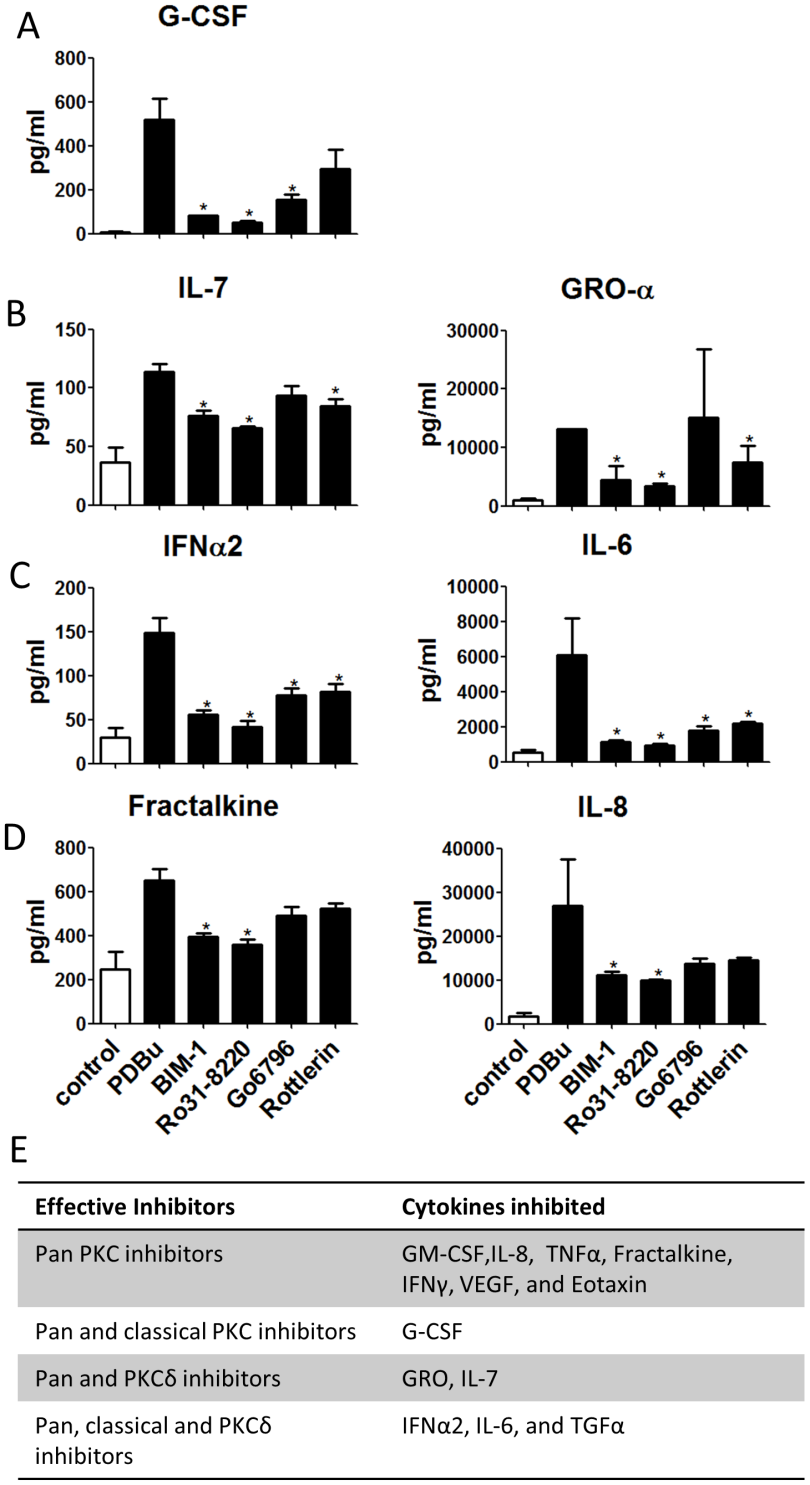
PDBu-induced cytokines were selectively regulated by different PKC sub-class isozymes. (A) G-CSF was inhibited by pan or classical PKC inhibitors. (B) IL-7 and GRO-1α were inhibited by pan PKC or novel PKC inhibitors. (C) IFNα2 and IL-6 are inhibited by pan, classical PKC or novel PKC inhibitors. (D) Fractalkine and IL-8 are inhibited only by pan PKC inhibitors. (E) The table summarizes the selective regulation of cytokines by different PKC subclass isozymes.

**Figure 7:**
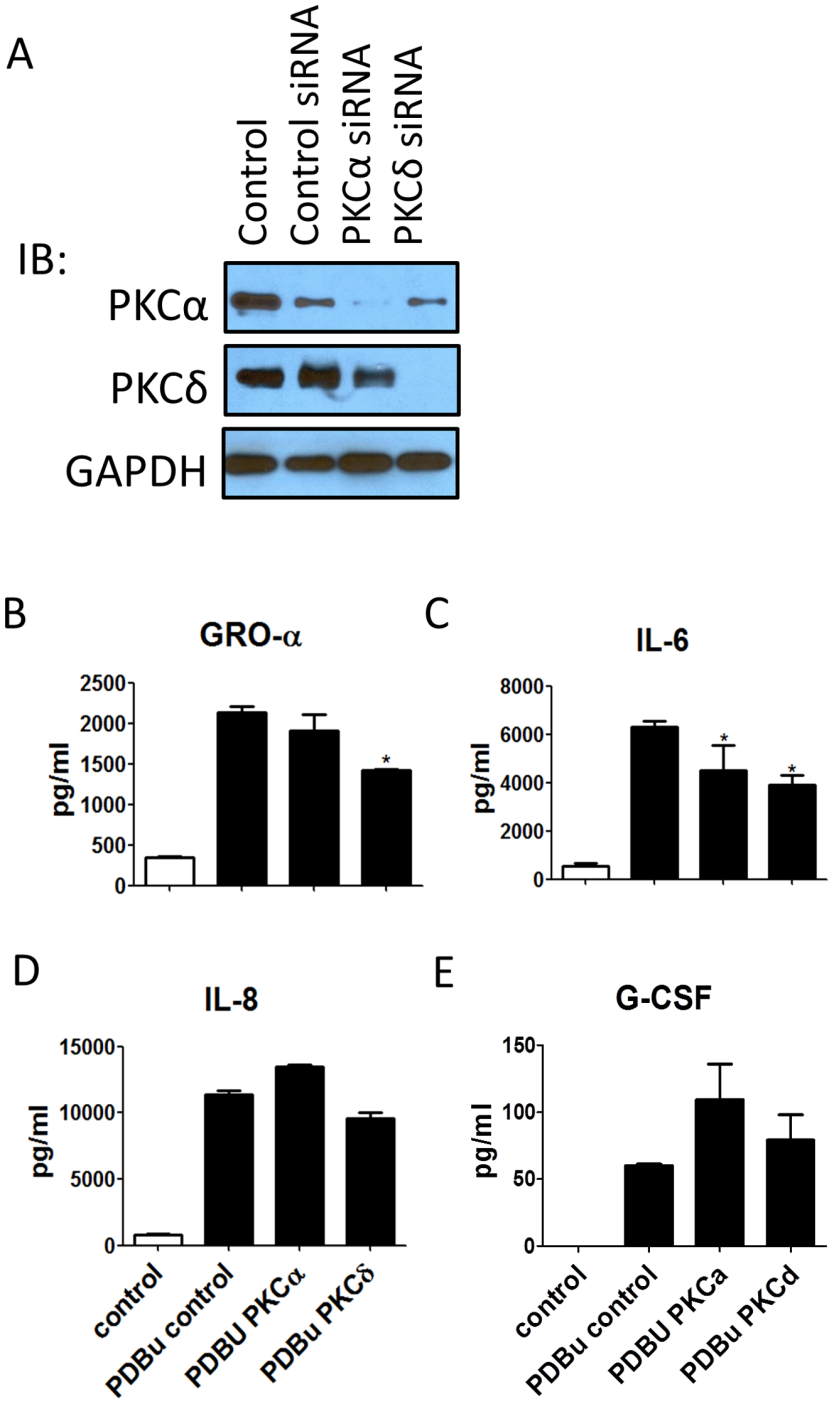
PKCα and PKCδ siRNA were utilized to confirm the inhibitor results. (A) The protein expressions of PKCα and PKCδ were specifically downregulated by their respective siRNAs. (B) PKCδ, but not PKCα siRNA, was able to inhibit the GRO-1α production. (C) Both PKCδ and PKCα siRNA inhibited the IL-6 production. (D) Neither PKCδ siRNA nor PKCα siRNA were able to inhibit the IL-8 production. (E) PKCα siRNA did not reduce the G-CSF production, suggesting that classical PKCs other than PKCα regulate the production of G-CSF.

All of the cytokines whose production is increased after PDBu stimulation were effectively inhibited by pan PKC inhibitors. G-CSF was significantly reduced by Gö6796, suggesting that classical PKC isozymes are involved in regulating its production (Figure 6A). GRO-1α and IL-7 were significantly blocked by rottlerin, suggesting that their production is regulated by novel PKCs (Figure 6B). IFNα2 and IL-6 were effectively inhibited by either classical or novel inhibitor, suggesting both of the PKC types are involved in the regulation of their production in a cooperative fashion (Figure 6C). In contrast, neither the classical nor the novel PKC inhibitor alone significantly reduced Fractalkine and IL-8 levels (Figure 6D), suggesting that both subclasses may independently regulate their production, thus, complete blockage requires blocking of both pathways. Figure 6E summarizes these findings. To confirm the results from cytometric beads, we then measured a few of the cytokines with ELISA. Furthermore, PKCα and PKCδ siRNA were used as examples to verify the roles of classical and novel PKC isozymes. Each of the siRNA specifically blocked the expression of their target PKC isoforms (Figure 7A). PKCδ but not PKCα siRNA reduced PDBu-induced GRO-1α (Figure 7B). Both PKCα and PKCδ siRNA, when treated alone, significantly reduced PDBu-induced IL-6 (Figure 7C). Moreover, neither PKCα nor PKCδ siRNA reduced PDBu-induced IL-8 (Figure 7D). These results are in agreement with the inhibitor data (Figure 6). However, PKCα downregulation was unable to decrease the PDBu-induced production of G-CSF, which contrasts with the inhibitor study (Figure 7E), suggesting that the production of G-CSF is mediated by other classical PKC isozymes.

## Discussion

Although many previous studies have demonstrated that PKC family can be activated by a variety of environmental substances and inflammatory cytokines and is involved in inflammation in the airway, the direct role of PKC in the inflammatory response is largely unknown. In the present study, we addressed this question by bioinformatics approach to determine the PKC-induced cellular responses in human bronchial epithelial cells. We examined the overall change in the gene and cytokine expression profile. Our microarray result shows that PKC activation elicits gene expression changes with multiple cellular functions, particularly genes related to cell death and inflammation. These changes were further manifested by increase in cell death and production of multiple cytokines. Furthermore, classical and novel PKCs regulate the production of various biologically important cytokines in a different fashion.

PKC activation effectively regulates gene expression in human bronchial epithelial cells. Microarray analysis suggests that PKC activation induces change in more than 500 genes. Most of the genes were up-regulated, indicating that the major effect of PKC activation is on gene transcription. Early response genes were up-regulated within the first 0.5 hours of PDBu stimulation. Proteins encoded by these genes are transcription factors that regulate other gene expression. Several genes encoding cytokines are also up-regulated immediately after the PKC activation, indicating that the immediate inflammatory response of airway epithelial cells can be turned on by PKC related mechanisms.

Results from Functional Annotation and Ingenuity Pathway studies support our hypothesis that PKC activation is involved in multiple cellular functions, which include gene expression, wound healing, cell proliferation, metabolic process, cellular movements and immune cell trafficking. However, the most dominant effects of PKC activation in airway epithelial cells are cytokine production and apoptosis. The top IPA interaction network is around NFκB complex, a well-known transcription factor mediating inflammatory responses by controlling gene expression of various pro-inflammatory cytokines, adhesion molecules, and matrix metalloproteinases [19]–[22]. This finding supports other previous observations that various pathogen-induced inflammatory responses are mediated through PKC and NFκB. For example, chitinase is an enzyme produced by a variety of organisms such as insect, parasite, fungus, bacterium, plant and animal. Chitinase activates classical PKC, ERK and NFκB in sequence to synthesize and release IL-8, a pro-inflammatory chemokine, in lung epithelial cells [23]. Moraxella catarrhalise, a bacterial species that exacerbates chronic obstructive lung disease, can also induce IL-8 production by activating PKCα, ε, and θ to augment NFκB-regulated transcription [24]. TNF-α, another important molecule suggested by the interaction network, is a well-known cytokine that initiates inflammation, immune response and cell death [25]–[27]. It has been reported that TNF-α induced ICAM-1 expression in human airway epithelial cells is mediated through a pathway regulated by PKC and NFκB [28]. The functional annotations revealed that genes related to both apoptosis and cell proliferation are turned on by PKC activation. When cells are under stress, both death anti-death signals can be activated. The balance between these signals ultimately determines the fate of the cells. For instance, NFκB has been suggested to play a dichotomous role in apoptosis by either inducing cell death through up-regulating FasL production, or by inhibiting cell death through suppression of caspase-8 and regulating Akt pathways [29]–[31]. Also studies have suggested that TNFα may also play a role in cell proliferation and anti-apoptosis. Thus we further validated PKC activation modulation of cell survival via two separate tests that determines cell viability. Among different pathways of programmed cell death, Caspase mediated cell death is the most prevalent in many biological systems [32], [33]. We found that cleaved caspase-3 increased significantly after 4 hours of PKC activation. The number of viable cells significantly reduced 6 hours after PKC activation. This confirms that PKC activation leads to programmed cell death as suggested by the microarray analysis.

As demonstrated by CBA, human bronchial epithelial cells can produce various cytokines, chemokines and growth factors. Most cytokines levels were elevated by PKC activation, and almost all of the increased cytokines are pro-inflammatory. All of the top elevated cytokines (i.e., IL-6, IL-8, GM-CSF, IFNγ) are related to innate immunity, indicating PKC activation is crucial for the initial defence mechanism in the airway against pathogens [34]–[38]. Notably, one of the growth factors that were significantly up-regulated after PKC activation is PDGF. PDGF has been reported to be secreted in response to thrombin and induces lung and airway remodelling [39]. We also found although most of cytokines can be increased by PKC activation at both mRNA and protein levels, some of the cytokines were elevated only at the mRNA level, while some others only at protein level. Inhibitor study suggests that these cytokines and chemokines are differentially regulated by sub-class PKC isozymes. Although these studies are preliminary, it shows the complexity of inflammatory responses, whereby each inflammatory cytokine is regulated by different pathways. It also demonstrates that the functional genomic studies are powerful tools allowing one to examine the overall mechanisms in a comprehensive approach.

In summary, PKC activation alone initiates gene expression related to multiple cellular functions, especially to cell death and inflammatory responses in airway epithelial cells. The fact that different PKC isozymes differentially regulate various cytokines at mRNA and/or protein levels shows the complexity of regulation on inflammatory responses. Further studies can elucidate these mechanisms and their specific downstream pathways to help develop new therapies for airway diseases related to inflammation induced by different pathogens or acute immune response.

## Supporting Information

Figure S1 PDBu treatment (500 nM) rapidly induced translocation of PKCα from cytosol to membrane fraction (top) and increased phosphorylation of various PKC isozymes (bottom).
(TIF)

Table S1 The primers sequence used for qRT-PCR to validate microarray analysis.
(TIF)
