# Supplementary figures and images for: PKC Activation Induces Inflammatory Response and Cell Death in Human Bronchial Epithelial Cells
Source: PLoS One. 2013 May 17;8(5):e64182. doi: 10.1371/journal.pone.0064182 (PMC3656947; doi:10.1371/journal.pone.0064182)

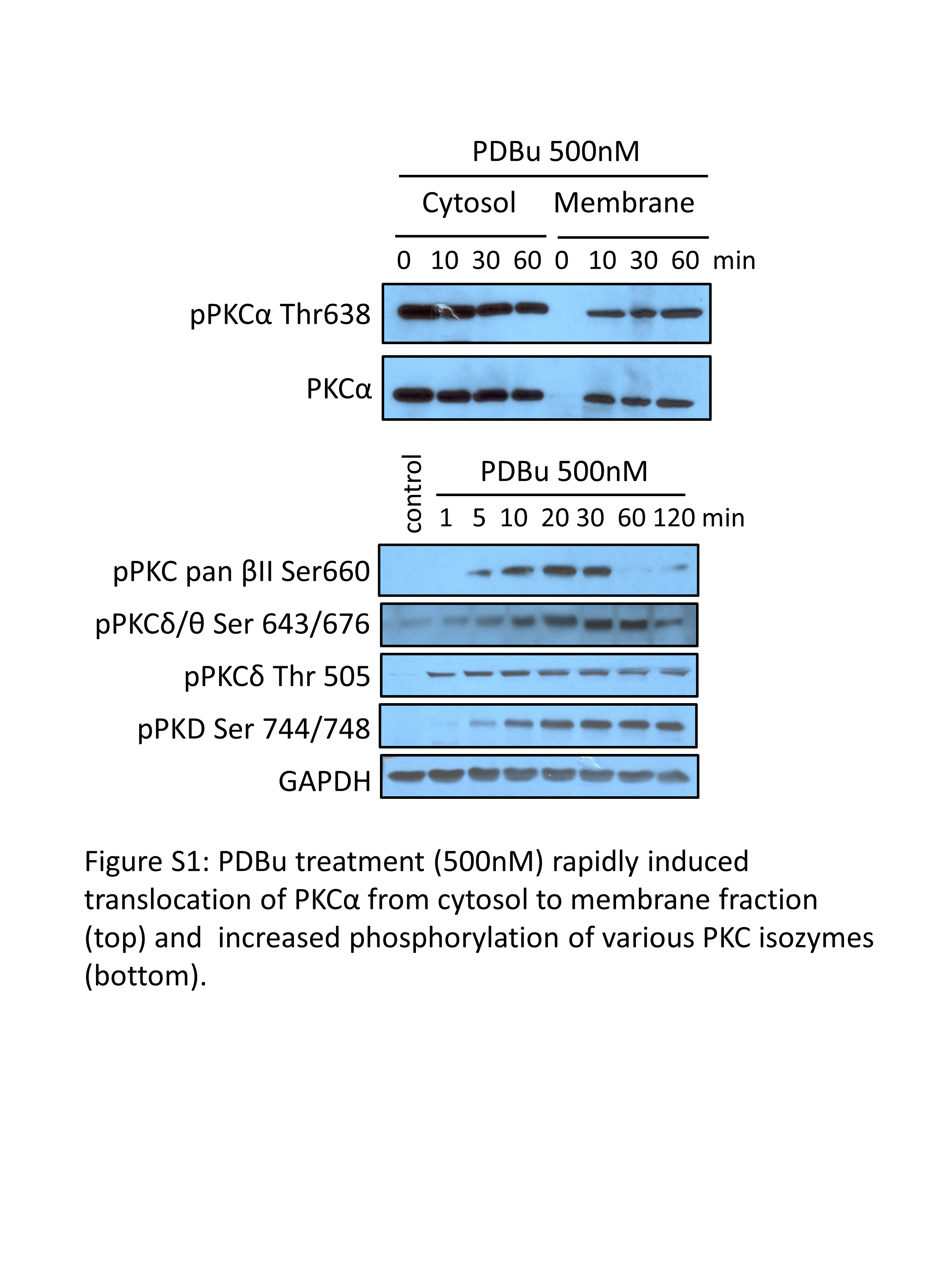

Supplement: Figure S1 — PDBu treatment (500 nM) rapidly induced translocation of PKCα from cytosol to membrane fraction (top) and increased phosphorylation of various PKC isozymes (bottom). (TIF) [file pone.0064182.s001.tif]

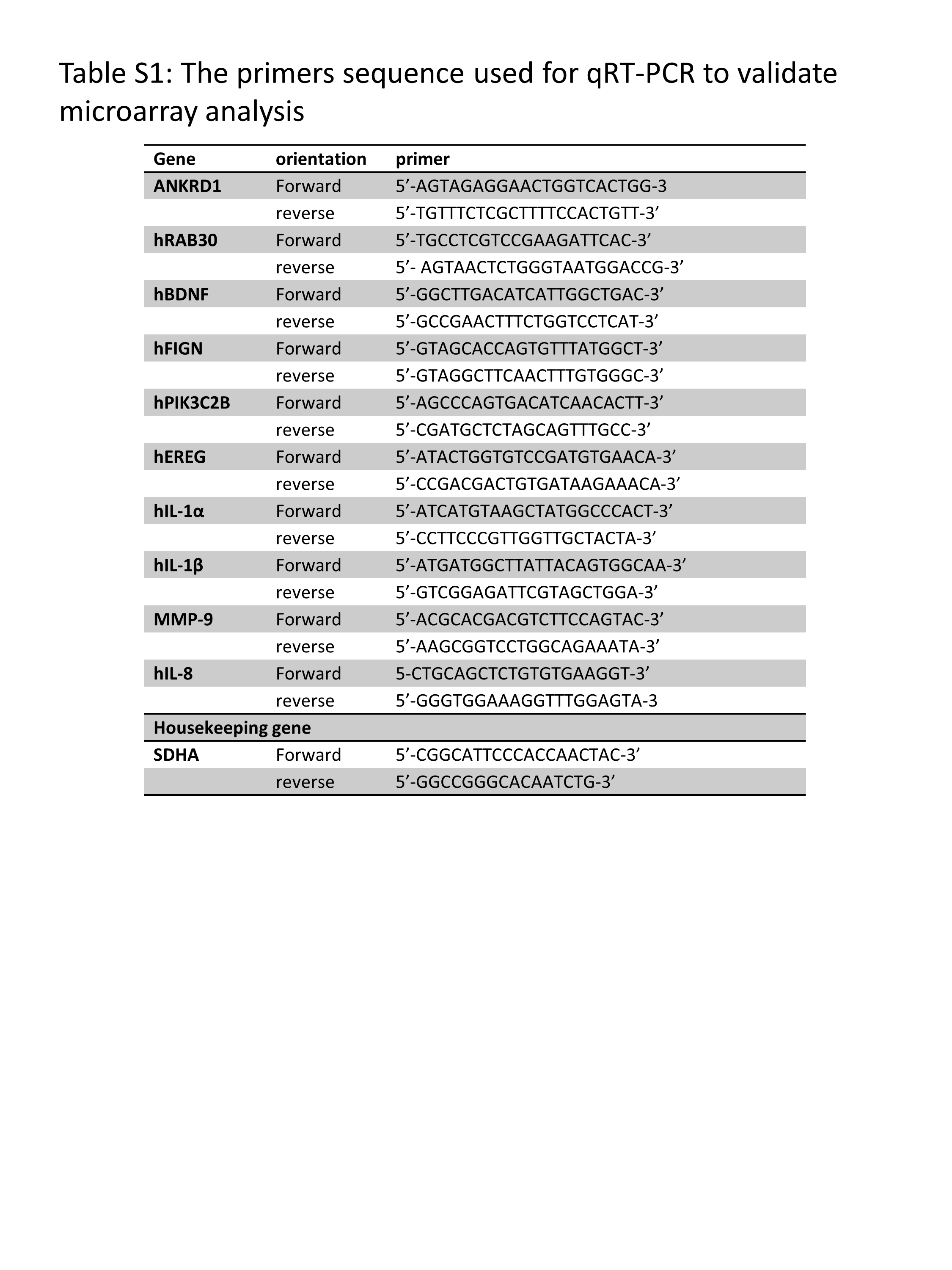

Supplement: Table S1 — The primers sequence used for qRT-PCR to validate microarray analysis. (TIF) [file pone.0064182.s002.tif]
